# Factors associated with syphilis treatment failure and reinfection: a longitudinal cohort study in Shenzhen, China

**DOI:** 10.1186/s12879-017-2715-z

**Published:** 2017-09-13

**Authors:** Zhenzhou Luo, Lin Zhu, Yi Ding, Jun Yuan, Wu Li, Qiuhong Wu, Lishan Tian, Li Zhang, Guomao Zhou, Tao Zhang, Jianping Ma, Zhongwei Chen, Tubao Yang, Tiejian Feng, Min Zhang

**Affiliations:** 1 Department of Dermatology and Venereology, Nanshan Center for Chronic Disease Control, Shenzhen, 518054 China; 2 Sexually Transmitted Disease Clinic, Xili People’s Hospital, Shenzhen, China; 3 School of Public Health, Central South University, Changsha, China; 4 Shenzhen Center for Chronic Disease Control, Shenzhen, China

**Keywords:** Syphilis, Treatment failure, Reinfection, Cohort study

## Abstract

**Background:**

The treatment failure and reinfection rates among syphilis patients are high, and relevant studies in China are limited. The aim of this study was to detect the rates of treatment failure and reinfection after syphilis treatment and to explore the potential associated factors.

**Methods:**

We conducted a longitudinal cohort study in a sexually transmitted disease clinic, the Department of Dermatology and Venereology in Nanshan Center for Chronic Disease Control. Serological testing was performed at baseline and throughout the 2-year follow-up for syphilis patients. To identify potential predictors of treatment outcomes, multivariate logistics analyses were utilized to compare the demographic and clinical characteristics of patients with serological failure/reinfection to those with serological cure/serofast.

**Results:**

From June 2011 to June 2016, a total of 1133 patients were screened for syphilis. Among the 770 patients who completed the 2-year follow-up, 510 first-diagnosed patients were included in the final analysis. Multivariate logistics analysis revealed the stage of syphilis (secondary syphilis VS. primary syphilis: adjusted odds ratio, 3.50; 95% confidence interval, 1.11-15.47; *p* = 0.04), HIV status (positive VS. negative: adjusted odds ratio, 3.06; 95% confidence interval, 1.15-8.04; *p* = 0.02) and frequency of condom use (always use VS. never use: adjusted odds ratio, 0.28; 95% confidence interval 0.08-0.75; p = 0.02) were significantly associated with the serological outcome.

**Conclusions:**

The clinical implications of our findings suggest that it is very important to perform regular clinical and serologic evaluations after treatment. Health counseling and safety education on sex activity should be intensified among HIV-infected patients and secondary syphilis patients after treatment.

## Background

Syphilis is a sexually transmitted disease (STD) caused by Treponema pallidum. Syphilis remains a common disease worldwide, and China has been burdened with the prevention, control and management of syphilis [1]. While syphilis could lead serious complications when left untreated, it is simple to cure with the appropriate treatment at early stages [2]. Having syphilis once does not protect patients from getting the disease again. Even after successful treatment, patients might still be re-infected by unprotected sexual contact. The re-infection rate of syphilis is high, especially among high-risk population [3–5]. For example, some studies in men who have sex with men (MSM) reported reinfection rates among this population ranging from 5.9 to 22% [6–9]. Two American studies also showed that 9.0 and 21.8% of human immunodeficiency virus (HIV) co-infected patients experienced syphilis treatment failure or re-infection, respectively [10, 11].

Although some researchers studied the response to treatment by serological monitoring after therapy, the focus of those studies was mainly on serological cure and serofast status [12, 13]. Studies on syphilis treatment failure and/or re-infection in China are limited. A better understanding of syphilis treatment failure and/or re-infection and the associated factors would help in identifying high-risk populations and improving treatment outcomes. Moreover, the results could provide scientific evidence for formulating and optimizing intervention strategies for syphilis.

In April 2011, Nanshan district, Shenzhen, launched the Syphilis Convergence Case-management Project. This pilot project aimed to consolidate prevention, treatment and management to better control syphilis. All the medical and public health faculties within the district are encouraged to provide syphilis seropositive cases referrals to the STD clinic, Department of Dermatology and Venereology in Nanshan Center for Chronic Disease Control, which is responsible for providing standardized treatment and regular serologic follow-up. We conducted a cohort study to compare the outcomes of treatment. The aim of our study was to detect the rates of treatment failure and re-infection and to explore the potential associated factors.

## Methods

### Study design and ethics statement

All subjects in the study were collected from the STD clinic, Department of Dermatology and Venereology, Nanshan Center for Chronic Disease Control, between June 2011 and June 2016. The diagnosis was confirmed by the Treponema Pallidum Particle Assay (TPPA) and Toludine Red Unheated Serum Test (TRUST), and TRUST was used to monitor the change in serological titers during follow-up and to subsequently judge the treatment outcome. Demographic characteristics, clinical characteristics (i.e., STI history, syphilis stage, TRUST titer), and behavioral characteristics (i.e., sexual orientation, number of sex partners, frequency of condom use in past 6 months) were collected at baseline. Disease stage was classified on the basis of clinical examination and medical history. Patients were considered to have primary syphilis (ulcer at anogenital or oropharyngeal sites and positive serology), secondary syphilis (mucocutaneous and/or skin lesions typical for syphilis and positive serology), or latent syphilis (no clinical signs of syphilis and positive serology). Neurosyphilis cases were excluded from the analysis.

All patients who were not allergic to penicillin received benzathine penicillin G 2.4 million units intramuscularly once weekly for three consecutive weeks. Penicillin allergic patients received doxycycline 100 mg twice daily orally for 30 days [14, 15]. After treatment, the patients had TRUST preformed once every three months in the first year (at 3, 6, 9 and 12 months) and once every six months in the second year (at 18 months and 24 months). All the testing was performed by the laboratory at the Nanshan Center for Chronic Disease Control.

This study was approved by the Institutional Ethics Committee of Nanshan Center for Chronic Disease Control and was in compliance with the national legislation and the Declaration of Helsinki guidelines. Written patient consent was obtained according to the institutional guidelines.

### Study outcomes

The primary outcome was response to syphilis treatment, determined on the basis of change in TRUST titers after treatment. TRUST titers were assessed and categorized into serological cure/serofast and treatment failure/reinfection. Serological cure was defined as either negative TRUST test results or a > 4-fold (2 dilution) decrease in the titer within 12 months and no titer pattern consistent with re-infection during the subsequent follow-up. After one year of recommended therapy, syphilis patients were considered to be in a serofast state if their TRUST test remained positive and the titers neither increased nor decreased by at least four-fold. Serological failure was defined as a lack of a 4-fold decrease in TRUST titers after the initial treatment or a titer pattern consistent with re-infection. The pattern was characterized as a decrease in TRUST titers after treatment but subsequent increase 4-fold compared with that of the last follow-up.

### Data analysis

To identify potential predictors of developing serologic failure, we compared the demographic and clinical characteristics of patients with serological failure/reinfection to patients with serological cure/serofast using bivariate logistics analysis. The odds ratios (OR) were estimated with 95% confidence intervals (CIs) from the bivariate analysis, and factors with *p* < 0.2 were further included in the multivariate analysis [12, 13, 16]. Adjusted odds ratios (AOR) with 95% confidence intervals were estimated from the regression analysis. All statistical analyses were conducted using R software (version 3.3.0), and *p* < 0.05 (two sided) was considered a statistically significant difference.

## Results

**Fig. 1:**
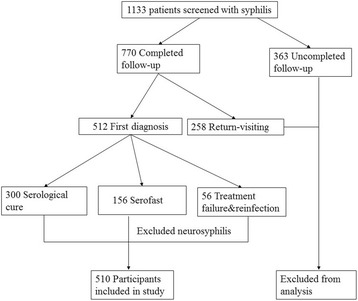
Flow of participants in the study and inclusion in the analysis

During the study period, 1133 patients were screened for syphilis and received standard treatment. Among the 770 patients who completed the 2-year follow-up, 512 were first diagnosed. After excluding two neurosyphilis cases, 510 participants were included in the analysis (Fig. 1).

The proportions of evaluable participants who exhibited effective treatment varied by syphilis stage and time point after therapy. Overall, the effective proportion of treatment was 89.06% (456/510), which included 300 (58.60%) serological cures and 156 (30.46%) serofasts. A total of 56 (10.94%) participants experienced treatment failure or reinfection, and the proportion of MSM population, HIV-infected participants and combined patients were 18.03, 15.78 and 18.18%, respectively.

We compared the characteristics of the 510 participants with effective treatment to the characteristics of participants with treatment failure and reinfection within the two-year follow-up (Table 1). The median ages of the effective treatment group and treatment failure group were 33.0 and 33.5, respectively. Females were associated with increased odds of treatment failure and reinfection. The stage of syphilis was also detected through the bivariate analysis. The odds ratio for secondary syphilis patients was 3.14 (95% CI: 1.02-13.73) compared with primary syphilis patients. Having a baseline TRUST titer ≥1:32 was associated with a decreased probability of being cured compared with having a baseline TRUST titer >1:32. The HIV-infected and MSM population showed an increased trend of experiencing treatment failure or reinfection. A decreased odds of treatment failure was shown for participants who always use a condom in the past 6 months compared with the participants who never use a condom. Age, history of STI, and number of sex partners were not significantly associated with response to treatment.

**Table 1:** Bivariate analysis of characteristics associated with treatment failure and reinfection

Characteristic	Serological cure/Serofast	Treatment failure/Reinfection	OR (95% CI)	*p-*value
Age				
< 25	100	9	–	
25-35	154	22	1.26 (0.73, 2.30)	0.42
> 35	200	25	0.78 (0.47, 1.29)	0.33
Gender				
Male	285	29	–	
Female	169	27	1.57 (0.89, 2.74)	0.11
Syphilis stage				
primary	61	3	–	
secondary	123	19	3.14 (1.02, 13.73)	0.07
latent	270	34	2.56 (0.88, 10.87)	0.13
Baseline titer				
< 1:32	289	27	–	
≥ 1:32	165	29	1.88 (1.07, 3.30)	0.02
HIV status				
Positive	50	11	1.98 (0.92, 3.95)	0.06
Negative	404	45	–	
Sexual orientation				
Non-MSM	390	44	–	
MSM	64	12	1.66 (0.80, 3.22)	0.15
History of STIs^a^				
No	398	52	–	
Yes	56	4	0.54 (0.16, 1.40)	0.26
No. of sex partners				
0-2	352	44	–	
3-5	62	5	0.64 (0.21, 1.55)	0.37
6-10	25	4	1.28 (0.36, 3.49)	0.66
> 10	15	3	1.60 (0.36, 5.09)	0.47
Frequency of condom use				
Never	272	38	–	
Sometimes	88	11	0.89 (0.42, 1.77)	0.76
Always	94	7	0.53 (0.22, 1.16)	0.14

^a^History of STI includes herpes progenitalis, chancroid, trichomoniasis, chlamydia, gonorrhea, and condyloma acuminate

A multivariate analysis was then conducted using all the significant variables identified above. As shown in Table 2, the results further confirmed that the stage of syphilis, HIV status and frequency of condom use were significantly associated with treatment failure and reinfection. The adjusted odds ratio (AOR) for secondary syphilis patients was 3.49 (95% CI: 1.11, 15.47 *P* = 0.04). HIV co-infection was associated with a 3-fold increased odds of treatment failure or reinfection (AOR, 3.05; 95% CI, 1.14-8.04; *p* = 0.02). In addition, the odds ratio for patients who always use a condom reduced 72% compared with the patients who never use a condom (AOR, 0.28; 95% CI 0.08-0.75; *p* = 0.02).

**Table 2:** Adjusted odds ratios and 95% confidence intervals for characteristics associated with treatment failure and reinfection

Parameter	AOR^a^(95% CI)	*p-*value
Syphilis stage		
Secondary VS Primary	3.50 (1.11, 15.47)	0.04
Latent VS Primary	2.54 (0.85, 10.96)	0.14
HIV status		
Positive VS Negative	3.06 (1.15, 8.04)	0.02
Frequency of condom use		
Sometimes VS Never	0.79 (0.33 1.73)	0.57
Always VS Never	0.28 (0.08,0.75)	0.02

^a^Odds ratio was adjusted by gender, baseline titer and sexual orientation

## Discussion

Previous research has discussed the serological response to syphilis treatment [8–11, 14]. The time point used in the analysis is important when assessing the treatment effect, and a relatively wide window of time is recommended for determining serologic responses [2]. Compared with previous studies, each participant included in the final analysis received a 2-year follow-up, which provided more sufficient information to determine their outcomes. We report an overall rate of 10.94% among first-diagnosed patients, and the figures for the MSM population and HIV-infection patients were much higher. Therefore, it is very important to perform regular clinical and serologic evaluations after treatment, especially for the high-risk populations. To the best of our knowledge, this is the first study in China to detect the treatment failure and reinfection rate among STD clinic patients after long term follow-up.

We identified three potential associated factors with the treatment failure and reinfection in this study. The finding supports the findings of other investigations that also demonstrated that HIV- infected patients with syphilis might be more likely to experience serological failure and reinfection compared to non-HIV infected patients [17–19]. We speculate that HIV-infected patients with more immunosuppression might respond with a low rate to effective treatment. In addition, HIV might accelerate and change the clinical course of syphilis, and this co-infection could increase the incidence of the complications of syphilis [16, 20, 21]. Furthermore, HIV makes it more likely for syphilis to present with non-typical features [2]. For example, a UK enhanced surveillance program reported on the presentation of syphilis both in HIV-negative and HIV-positive men. Primary syphilis was diagnosed in 42 and 27%, and secondary disease was diagnosed in 40 and 58% of HIV-negative and HIV-positive patients, respectively [22]. Therefore, it is important for medical practitioners to be aware of how syphilis might present in patients with underlying HIV infection and the implications for treatment and follow-up.

We also found that secondary syphilis patients are more likely to experience treatment failure or reinfection compared with primary syphilis patients. Although there are no concrete indications regarding the different stages, and the clinical stages of syphilis often overlap, this finding highlights the importance of early diagnosis and treatment. Furthermore, the frequency of condom use was found to be associated with the response to treatment. Patients might be linked to recurrent exposure of syphilis from their sexual networks through unprotected sexual activity [23]. Patients who practice safe sex are less likely to be re-exposed to risky partners. Unprotected sexual behaviors, therefore, increase the likelihood of experiencing treatment failure or reinfection. This finding suggested that health counseling and safety education on sex activity should be intensified to increase condom use, especially among high risk populations.

Our study has several limitations. First, selection bias is possible because 28% of patients were excluded due to lack of data or loss of follow-up. If the exclusion of these patients affected the outcome is unknown. Second, some information collected at baseline is retrospective, such as the number of sex partners and the frequency of condom use, which might not reflect the actual conditions during the follow-up. In addition, a relatively rough classification was adopted in this study. We combined serological cure and serofast together. We did not distinguish patients with treatment failure or reinfection in the analysis, so it is hard to make a distinction without information regarding their exposure and network. Furthermore, the bivariate analysis used for the first-step variable selection might be not biologically plausible all of the time. Additionally, the results from one city might be not well generalized to other regions. However, we believe this study provides a good representation of southeast coastal cities, and more studies in diverse areas are needed in further research.

## Conclusions

Treatment failure and reinfection of syphilis should be considered in the management of syphilis patients. The clinical implications of our findings suggest that HIV-infected patients and secondary syphilis patients should be closely monitored for serological failure after syphilis treatment.

## Abbreviations

AOR: Adjusted odds ratios
CI: Confidence intervals
HIV: Human immunodeficiency virus
MSM: Disease men who have sex with men
OR: Odds ratios
STD: Sexually transmitted
TPPA: Treponema Pallidum Particle Assay
TRUST: Toludine Red Unheated Serum Test
